# Supplementary material for: Transient protein accumulation at the center of the T cell antigen-presenting cell interface drives efficient IL-2 secretion
Source: eLife. 2019 Oct 30;8:e45789. doi: 10.7554/eLife.45789 (PMC6821493; doi:10.7554/eLife.45789)
Supplement: Figure 1—source data 1. — Publications describing the sensors used in Figure 1C,D and representative 5C.C7 T cell imaging data are given. [file elife-45789-fig1-data1.pdf]

Figure 1–figure supplement 5

**Signaling intermediate**	**Sensor**	**Location of classification data/representative video**
**Actin**	GFP-Actin	J. Immunol. (2003) *171,* 2287-95
**Akt**	Akt-GFP	PLoS One (2015) *10,* e0133299
**Arp3**	Arp3-GFP	Sci. Signal. (2016) *9*, rs3
**Capping protein 1 α**	Capping protein 1 α-GFP	Sci. Signal. (2016) *9*, rs3
**CD2**	CD48-GFP	Sci. Signal. (2009) *2*, ra15
**Cofilin**	Cofilin-GFP	Sci. Signal. (2016) *9*, rs3
**Coronin 1A**	Coronin 1A-GFP	Sci. Signal. (2016) *9*, rs3
**Ezrin**	Ezrin-GFP	PLoS One (2015) *10,* e0133299
**Grb2**	Grb2-GFP	PLoS One (2015) *10,* e0133299
**HS1**	HS1-GFP	Sci. Signal. (2016) *9*, rs3
**Itk**	Itk-GFP	PLoS One (2015) *10,* e0133299
**LAT**	LAT-GFP	Sci. Signal. (2009) *2*, ra15
**Lck**	Lck-GFP	PLoS One (2015) *10,* e0133299
**Myosin II RLC**	Myosin II RLC-GFP	Sci. Signal. (2016) *9*, rs3
**NFκB p65**	GFP-p65	PLoS One (2015) *10,* e0133299
**PIP_2**	GFP-PLCδ-PH	PLoS One (2015) *10,* e0133299
**PKC Θ**	PKC Θ-GFP	Sci. Signal. (2009) *2*, ra15
**SLP-76**	SLP-76-GFP	PLoS One (2015) *10,* e0133299
**TCR as TCRζ**	TCRζ-GFP	Sci. Signal. (2009) *2*, ra15
**Vav1**	Vav1-GFP	PLoS One (2015) *10,* e0133299
**WASP**	GFP-WASP	Sci. Signal. (2016) *9*, rs3
**WAVE-2**	GFP-WAVE2	Sci. Signal. (2016) *9*, rs3
