# Supplementary figures and images for: Transient protein accumulation at the center of the T cell antigen-presenting cell interface drives efficient IL-2 secretion
Source: eLife. 2019 Oct 30;8:e45789. doi: 10.7554/eLife.45789 (PMC6821493; doi:10.7554/eLife.45789)

**A**

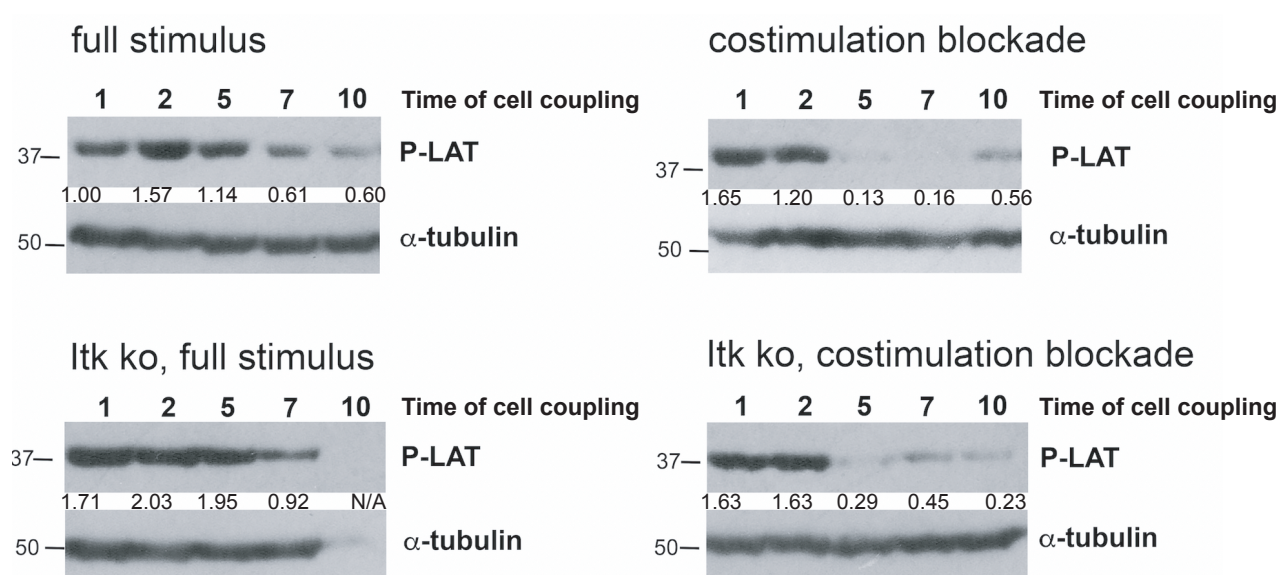

**B**

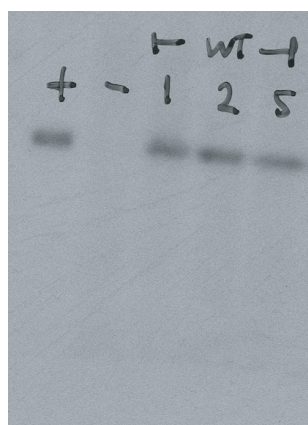

Supplement: Figure 2—source data 1. — No entry indicates p>0.05. 0.000 indicates p<0.0005. Gray scale is used to visualize the level of significance. [file elife-45789-fig2-data1.pdf]
