# Supplementary material for: Transient protein accumulation at the center of the T cell antigen-presenting cell interface drives efficient IL-2 secretion
Source: eLife. 2019 Oct 30;8:e45789. doi: 10.7554/eLife.45789 (PMC6821493; doi:10.7554/eLife.45789)
Supplement: Figure 4—source data 1. — No entry indicates p>0.05. 0.000 indicates p<0.0005. Gray scale is used to visualize the level of significance. [file elife-45789-fig4-data1.pdf]

Figure 4–figure supplement 2

LAT V3

Condition	Comparison	Pattern	-40	-20	0	20	40	60	80	100	120	180	300	420
**Restoration to LAT full stimulus**														
LAT V3, full stimulus	LAT, full stimulus	any			0.03	0.001	0.000	0.000	0.000	0.000	0.000	0.000	0.000	0.000
		central					0.004	0.001	0.000	0.000	0.000	0.000	0.03	0.002
LAT V3, Itk ko, full stimulus	LAT, full stimulus	any		0.002			0.04	0.01	0.004	0.003	0.002	0.001	0.001	0.000
		central			0.01		0.007	0.006	0.000	0.000	0.000	0.000	0.004	0.006
LAT V3, anti-B7	LAT, full stimulus	any		0.006		0.008	0.002	0.004	0.003	0.000	0.000	0.000	0.006	0.006
		central					0.04	0.03	0.02	0.000	0.000	0.000		
LAT V3, Itk ko anti-B7	LAT, full stimulus	any			0.000	0.006						0.01	0.009	0.02
		central			0.000		0.000	0.000	0.000	0.000	0.000	0.000	0.03	0.003
**Enhancement under matched stimuli**														
LAT V3, Itk ko, full stimulus	LAT, Itk ko, full stimulus	any		0.004	0.007				0.03	0.01	0.004	0.04	0.01	0.001
		central				0.03	0.01	0.005	0.001	0.000	0.001	0.001	0.006	0.004
LAT V3, anti-B7	LAT, anti-B7	any					0.002	0.001	0.000	0.000	0.000	0.000	0.006	0.003
		central				0.02	0.000	0.000	0.000	0.000	0.000	0.000	0.003	0.004
LAT V3, Itk ko, anti-B7	LAT, Itk ko, anti-B7	any		0.000	0.000	0.000	0.000	0.000	0.000	0.000	0.000	0.000	0.001	0.000
		central			0.04	0.001	0.000	0.000	0.000	0.000	0.000	0.000	0.01	0.000

LAT Vav

Condition	Comparison	Pattern	-40	-20	0	20	40	60	80	100	120	180	300	420
**Restoration to LAT full stimulus**														
LAT Vav, full stimulus	LAT, full stimulus	any			0.002	0.03	0.004	0.002	0.001	0.003	0.000	0.002		
		central			0.05	0.01								
LAT Vav, Itk ko, full stimulus	LAT, full stimulus	any			0.000	0.001							0.008	
		central			0.005			0.001	0.001	0.000	0.000	0.001		
LAT Vav, anti-B7	LAT, full stimulus	any												
		central					0.002	0.002	0.002	0.000	0.002			
LAT Vav, Itk ko anti-B7	LAT, full stimulus	any			0.000	0.000						0.04		
		central			0.000		0.02	0.006	0.000	0.000	0.000	0.002		0.05
**Enhancement under matched stimuli**														
LAT Vav, Itk ko, full stimulus	LAT, Itk ko, full stimulus	any				0.01	0.05						0.05	
		central						0.001	0.05	0.02	0.03	0.005		
LAT Vav, anti-B7	LAT, anti-B7	any			0.001	0.01	0.000	0.000	0.001	0.001	0.005			0.03
		central			0.000	0.000	0.000	0.000	0.001	0.000	0.000			
LAT Vav, Itk ko, anti-B7	LAT, Itk ko, anti-B7	any				0.03	0.001	0.000	0.000	0.000	0.000	0.000	0.001	0.000
		central				0.005	0.000	0.000	0.000	0.000	0.000	0.000	0.03	0.007

LAT PLCdPH

Condition	Comparison	Pattern	-40	-20	0	20	40	60	80	100	120	180	300	420
**Restoration to LAT full stimulus**														
LAT PLCd, full stimulus	LAT, full stimulus	any			0.000	0.000	0.000	0.002	0.03	0.03				
		central			0.000	0.05								
LAT PLCd, Itk ko, full stimulus	LAT, full stimulus	any			0.000	0.01			0.001		0.03			
		central			0.001				0.05	0.04				
LAT PLCd, anti-B7	LAT, full stimulus	any				0.03	0.01	0.002	0.000	0.000	0.000	0.000	0.001	0.05
		central				0.009								0.04
LAT PLCd, Itk ko anti-B7	LAT, full stimulus	any			0.000	0.009						0.005		
		central			0.001	0.02								
**Enhancement under matched stimuli**														
LAT PLCd, Itk ko, full stimulus	LAT, Itk ko, full stimulus	any							0.005		0.05			
		central	0.05											
LAT PLCd, anti-B7	LAT, anti-B7	any			0.009			0.03						
		central			0.01									
LAT PLCd, Itk ko anti-B7	LAT, Itk ko, anti-B7	any				0.005	0.002	0.000	0.000	0.000	0.000	0.000	0.002	0.002
		central								0.003	0.05			
