# Supplementary material for: Transient protein accumulation at the center of the T cell antigen-presenting cell interface drives efficient IL-2 secretion
Source: eLife. 2019 Oct 30;8:e45789. doi: 10.7554/eLife.45789 (PMC6821493; doi:10.7554/eLife.45789)
Supplement: Figure 6—source data 1. — No entry indicates p>0.05. 0.000 indicates p<0.0005. Gray scale is used to visualize the level of significance. [file elife-45789-fig6-data1.pdf]

Figure 6–figure supplement 1

Condition	Comparison	Pattern	-40	-20	0	20	40	60	80	100	120	180	300	420
Grb2 + LATV3, full stimulus	Grb2, full stimulus	any			0.01	0.000	0.003	0.001	0.000	0.02				
		central										0.003		
Grb2 + LATV3, Itk ko, full stim.	Grb2, Itk ko, full stim	any		0.03					0.02	0.03				
		central					0.04		0.001	0.001	0.01		0.009	
Grb2 + LATV3, anti-B7	Grb2, anti-B7	any		0.002	0.000	0.02	0.02		0.009				0.04	
		central	0.05	0.05								0.04		
Grb2 + LATV3, Itk ko, anti-B7	Grb2, Itk ko, anti-B7	any				0.003	0.000	0.001	0.000	0.000	0.000	0.000	0.001	0.01
		central			0.03	0.001	0.000	0.001		0.007		0.008		
Lck + LATV3, full stimulus	Lck, full stimulus	any		0.04		0.02	0.04	0.001	0.001	0.001	0.000	0.000	0.000	0.001
		central		0.03		0.000	0.001	0.000	0.008	0.008	0.05		0.007	
Lck + LATV3, Itk ko, full stim.	Lck, Itk ko, full stim	any				0.02	0.000	0.001	0.04	0.02				0.02
		central					0.007	0.003						
Lck + LATV3, anti-B7	Lck, anti-B7	any	0.05											
		central	0.05			0.03								
Vav1 + LATV3, full stimulus	Vav1, full stimulus	any		0.000	0.000	0.000	0.006	0.009	0.03	0.001		0.02		
		central		0.002	0.002	0.02								
Vav1 + LATV3, Itk ko, full stim.	Vav1, Itk ko, full stim	any												
		central												
Vav1 + LATV3, anti-B7	Vav1, anti-B7	any		0.000	0.02		0.05	0.03	0.03					
		central		0.01		0.02								
