# Supplementary material for: Transient protein accumulation at the center of the T cell antigen-presenting cell interface drives efficient IL-2 secretion
Source: eLife. 2019 Oct 30;8:e45789. doi: 10.7554/eLife.45789 (PMC6821493; doi:10.7554/eLife.45789)
Supplement: Figure 7—source data 1. — No entry indicates p>0.05. 0.000 indicates p<0.0005. Gray scale is used to visualize the level of significance. [file elife-45789-fig7-data1.pdf]

Figure 7–figure supplement 1

To Figure 7 panel B, left column

Condition	Comparison	Pattern	-40	-20	0	20	40	60	80	100	120	180	300	420
SLP-76, anti-B7	SLP-76, full stimulus	any				0.005	0.000	0.000	0.000	0.000	0.001	0.002		
		central			0.01	0.005	0.02	0.005						0.01
SLP-76, Itk ko, full stim.	SLP-76, full stimulus	any		0.05		0.001	0.000	0.003	0.000	0.000	0.000	0.006		
		central			0.000	0.002	0.002	0.003	0.04	0.04	0.05			
SLP-76, Itk ko, anti-B7	SLP-76, full stimulus	any			0.03	0.003	0.001	0.001	0.008	0.003				
		central			0.000	0.006				0.04				

To Figure 7 panel B, middle and right columns

SLP-76 V3

Condition	Comparison	Pattern	-40	-20	0	20	40	60	80	100	120	180	300	420
**Restoration to SLP-76 full stimulus**														
SLP-76 V3, full stimulus	SLP-76, full stimulus	any		0.04		0.03								
		central												
SLP-76 V3, Itk ko, full stimulus	SLP-76, full stimulus	any	0.01	0.000	0.01						0.05		0.03	0.003
		central		0.03	0.03	0.04	0.02							
SLP-76 V3, anti-B7	SLP-76, full stimulus	any							0.000	0.000	0.000	0.005		
		central								0.05				
SLP-76 V3, Itk ko anti-B7	SLP-76, full stimulus	any					0.03	0.05	0.000	0.001		0.02		
		central			0.02		0.04							
**Enhancement under matched stimuli**														
SLP-76 V3, Itk ko, full stimulus	SLP-76, Itk ko, full stimulus	any		0.000	0.000	0.000	0.000	0.000	0.000	0.000	0.000	0.000	0.001	0.000
		central					0.05							
SLP-76 V3, anti-B7	SLP-76, anti-B7	any			0.008	0.008	0.005				0.02			
		central			0.002		0.03							
SLP-76 V3, Itk ko anti-B7	SLP-76, Itk ko, anti-B7	any	0.05											
		central				0.03	0.004							

SLP-76 Vav

Condition	Comparison	Pattern	-40	-20	0	20	40	60	80	100	120	180	300	420
**Restoration to SLP-76 full stimulus**														
SLP-76 Vav, full stimulus	SLP-76, full stimulus	any												
		central												
SLP-76 Vav, Itk ko, full stimulus	SLP-76, full stimulus	any		0.03				0.03	0.01	0.02				
		central		0.02			0.04		0.006	0.05				
SLP-76 Vav, anti-B7	SLP-76, full stimulus	any			0.01		0.04	0.003	0.000	0.000	0.001	0.000		
		central			0.04	0.006								0.03
SLP-76 Vav, Itk ko anti-B7	SLP-76, full stimulus	any		0.03				0.03	0.01	0.02				
		central		0.02			0.04		0.006	0.05				
**Enhancement under matched stimuli**														
SLP-76 Vav, Itk ko, full stimulus	SLP-76, Itk ko, full stimulus	any					0.02				0.04		0.02	
		central			0.003		0.007	0.03	0.006	0.004				
SLP-76 Vav, anti-B7	SLP-76, anti-B7	any			0.000	0.001								
		central			0.000	0.000	0.02	0.01						
SLP-76 Vav, Itk ko anti-B7	SLP-76, Itk ko, anti-B7	any	0.05		0.007	0.02	0.03	0.01						
		central			0.02	0.02					0.05			
