# Supplementary material for: Transient protein accumulation at the center of the T cell antigen-presenting cell interface drives efficient IL-2 secretion
Source: eLife. 2019 Oct 30;8:e45789. doi: 10.7554/eLife.45789 (PMC6821493; doi:10.7554/eLife.45789)
Supplement: Figure 8—source data 1. — No entry indicates p>0.05. 0.000 indicates p<0.0005. Gray scale is used to visualize the level of significance. [file elife-45789-fig8-data1.pdf]

Figure 8–figure supplement 1

To Figure 8 panel B, left column

Condition	Comparison	Pattern	-40	-20	0	20	40	60	80	100	120	180	300	420
Grb2, anti-B7	Grb2, full stimulus	any				0.001		0.02	0.02					
		central				0.04		0.05						
Grb2, Itk ko, full stim.	Grb2, full stimulus	any	0.03		0.001	0.000	0.000	0.000	0.000	0.008				
		central										0.01		
Grb2, Itk ko, anti-B7	Grb2, full stimulus	any			0.002	0.000	0.000	0.002	0.006	0.03				
		central			0.02	0.003	0.05	0.006						

To Figure 8 panel B, middle and right columns

Grb2 V3

Condition	Comparison	Pattern	-40	-20	0	20	40	60	80	100	120	180	300	420
**Restoration to Grb2 full stimulus**														
Grb2 V3, full stimulus	Grb2, full stimulus	any												0.02
		central				0.000								0.05
Grb2 V3, Itk ko, full stimulus	Grb2, full stimulus	any		0.02		0.000	0.004	0.004	0.002					0.02
		central						0.006						
Grb2 V3, anti-B7	Grb2, full stimulus	any												0.04
		central				0.001				0.04				
Grb2 V3, Itk ko anti-B7	Grb2, full stimulus	any			0.000	0.000	0.000	0.000	0.000	0.001				
		central			0.01	0.01		0.04						
**Enhancement under matched stimuli**														
Grb2 V3, Itk ko, full stimulus	Grb2, Itk ko, full stimulus	any		0.01									0.03	
		central						0.03					0.05	
Grb2 V3, anti-B7	Grb2, anti-B7	any												
		central	0.05	0.05						0.04				
Grb2 V3, Itk ko anti-B7	Grb2, Itk ko, anti-B7	any	0.04	0.02	0.04			0.02	0.02					
		central							0.04					

Grb2 Vav

Condition	Comparison	Pattern	-40	-20	0	20	40	60	80	100	120	180	300	420
**Restoration to Grb2 full stimulus**														
Grb2 Vav, full stimulus	Grb2, full stimulus	any	0.05	0.000	0.000		0.05			0.05	0.002	0.000	0.02	0.04
		central			0.01	0.03		0.04					0.05	
		periphery		0.000	0.000	0.000	0.000	0.000	0.000	0.000	0.000	0.000	0.000	0.005
Grb2 Vav, Itk ko, full stimulus	Grb2, full stimulus	any	0.03	0.000	0.001		0.02	0.04	0.01	0.000	0.000	0.000	0.002	
		central			0.02	0.001	0.03	0.01	0.03	0.04			0.05	
		periphery		0.000	0.000	0.000	0.000	0.000	0.000	0.000	0.000	0.000	0.007	0.04
Grb2 Vav, anti-B7	Grb2, full stimulus	any	0.04	0.000	0.000									
		central			0.01			0.05						
		periphery		0.001	0.000	0.000	0.000	0.000	0.000	0.02	0.01			
Grb2 Vav, Itk ko anti-B7	Grb2, full stimulus	any		0.000	0.01		0.05		0.03	0.01	0.001	0.007		0.04
		central		0.03	0.000	0.01	0.003		0.02	0.04		0.03	0.05	0.03
		periphery		0.002	0.000		0.000	0.000	0.000	0.000	0.000	0.000	0.04	0.03
**Enhancement under matched stimuli**														
Grb2 Vav, Itk ko, full stimulus	Grb2, Itk ko, full stimulus	any		0.000	0.000	0.000	0.000	0.000	0.000	0.000	0.000	0.000	0.000	
		central				0.02	0.02	0.05		0.05	0.04	0.03	0.05	0.05
		periphery		0.000	0.000	0.000	0.000	0.000	0.000	0.000	0.000	0.000	0.003	
Grb2 Vav, anti-B7	Grb2, anti-B7	any		0.002	0.000	0.000								
		central	0.05											0.05
		periphery		0.007	0.000	0.000	0.004	0.000	0.001	0.004	0.008			
Grb2 Vav, Itk ko anti-B7	Grb2, Itk ko, anti-B7	any		0.03	0.000	0.000	0.000	0.000	0.000	0.000	0.004	0.01		
		central		0.02	0.01	0.02	0.02	0.02	0.01	0.03	0.02	0.01	0.02	0.01
		periphery			0.001	0.000	0.000	0.000	0.000	0.000	0.000	0.009		0.04
